# Clinical prediction models and the multiverse of madness

**DOI:** 10.1186/s12916-023-03212-y

**Published:** 2023-12-18

**Authors:** Richard D. Riley, Alexander Pate, Paula Dhiman, Lucinda Archer, Glen P. Martin, Gary S. Collins

**Affiliations:** 1 College of Medical and Dental Sciences, Institute of Applied Health Research, University of Birmingham, Birmingham, B15 2TT UK; 2 National Institute for Health and Care Research (NIHR) Birmingham Biomedical Research Centre, Birmingham, UK; 3 Division of Informatics, Imaging and Data Science, Faculty of Biology, Medicine and Health, University of Manchester, Manchester, UK; 4 Centre for Statistics in Medicine, Nuffield Department of Orthopaedics, Rheumatology and Musculoskeletal Sciences, University of Oxford, Oxford, OX3 7LD UK

**Keywords:** Clinical prediction model, Instability, Variance, Risk prediction, Bootstrapping, Mean absolute prediction error (MAPE)

## Abstract

**Background:**

Each year, thousands of clinical prediction models are developed to make predictions (e.g. estimated risk) to inform individual diagnosis and prognosis in healthcare. However, most are not reliable for use in clinical practice.

**Main body:**

We discuss how the creation of a prediction model (e.g. using regression or machine learning methods) is dependent on the sample and size of data used to develop it—were a different sample of the same size used from the same overarching population, the developed model could be very different even when the same model development methods are used. In other words, for each model created, there exists a multiverse of other potential models for that sample size and, crucially, an individual’s predicted value (e.g. estimated risk) may vary greatly across this multiverse. The more an individual’s prediction varies across the multiverse, the greater the instability. We show how small development datasets lead to more different models in the multiverse, often with vastly unstable individual predictions, and explain how this can be exposed by using bootstrapping and presenting instability plots. We recommend healthcare researchers seek to use large model development datasets to reduce instability concerns. This is especially important to ensure reliability across subgroups and improve model fairness in practice.

**Conclusions:**

Instability is concerning as an individual’s predicted value is used to guide their counselling, resource prioritisation, and clinical decision making. If different samples lead to different models with very different predictions for the same individual, then this should cast doubt into using a particular model for that individual. Therefore, visualising, quantifying and reporting the instability in individual-level predictions is essential when proposing a new model.

## Background

The multiverse refers to the potentially infinite number of other universes besides our own, which may or may not be similar. Related concepts are multiple realities, parallel worlds, and alternate universes. Although the multiverse is hypothetical, it gains growing popularity in science-fiction novels and films such as *Spider-Man: Into the Spider-Verse* and *Doctor Strange and the Multiverse of Madness*.

The idea of an infinite number of different realities is reflected in the theory of probability and statistics, which acknowledges the variability across random samples of the same size taken from a particular population. Different samples may lead to different sample estimates (e.g. of the proportion of pregnant women diagnosed with pre-eclampsia): some samples and estimates may be similar to each other, but others very different. The smaller the sample size, the more varied and unstable different sample estimates will be [1].

### Into the multiverse of prediction models

A multiverse of different samples, and therefore different sample estimates, presents a unique challenge for research studies developing clinical prediction models to inform diagnosis or prognosis for individuals [2]. These studies use a sample of data from a chosen target population (e.g. women 20 weeks pregnant; men diagnosed with prostate cancer) to develop a model for predicting an outcome value (e.g. blood pressure) or estimating an outcome risk (e.g. 10-year mortality risk) in any individual from that target population. The model is created using approaches such as regression, random forests or deep learning, which map predictor values (features) to outcomes (labels) at the individual level. An example is the ISARIC model [3], for use in hospitalised adults with suspected or confirmed COVID-19 to estimate their risk of in-hospital clinical deterioration based on 11 predictors measured at admission.

**Fig. 1:**
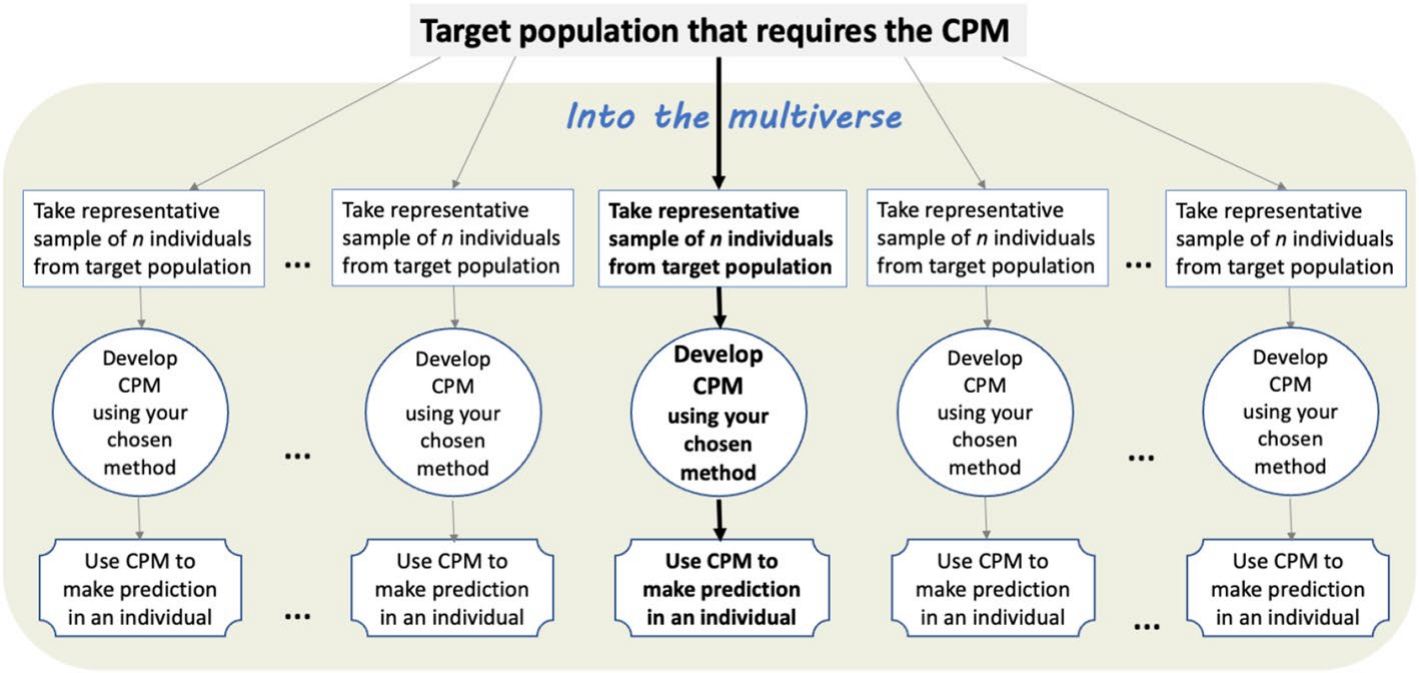
Depiction of the multiverse of clinical prediction models (CPMs) for a chosen target population. Each CPM is developed using the same model development method but from a different sample of size *n* from the target population of interest. After development, the CPM is used to make subsequent predictions for individuals. Bold arrows indicate the route that was actually taken, whilst grey arrows represent other hypothetical routes that would have been taken had a different dataset of size *n* been sampled

The creation of a prediction model is dependent on the sample and size of data used to develop it—were a *different sample of the same size *used from the same overarching population, the developed model might look very different (e.g. in terms of included predictors, predictor effects [4], regression equation, tuning parameters [5, 6], tree characteristics and splits) even when the same model development methods and set of candidate predictors are used. Therefore, whenever a prediction model is developed for a chosen target population, researchers must recognise there exists a multiverse of other potential models for the same prediction scenario (Fig. 1). The smaller the sample size, the more different the models in the multiverse will be and, crucially, the more varied their predicted values for the same individual. If the multiverse demonstrates large instability in predicted values for an individual, this implies that any one particular model is unlikely to be fit for purpose for that individual (e.g. to guide clinical decision making) [7]. This issue is rarely considered in current clinical prediction model research, as most models just provide a single predicted estimate for each individual (and any uncertainty of that estimate is ignored). It is strongly related to the concept of epistemic uncertainty (*reducible *uncertainty), which refers to uncertainty in predictions arising from the model production itself [8], rather than aleatoric uncertainty (*irreducible* uncertainty) that refers to residual uncertainty that cannot be explained by the model.

But what if we could examine instability by looking into the multiverse of models to see how different their predictions are—like the fourteen million, six hundred and five realities Dr Strange looked into at the end of the film, *Avengers Infinity War*? In this article, we explain how bootstrapping can be used to do this for a chosen target population [9–12] and argue that visualising, quantifying and reporting the instability in individual-level predictions should always be done during model development [13, 14].

## Main text

### Examining the multiverse using bootstrapping and instability plots

Clinical prediction model developers aim to produce models that minimise the variance (instability) of predictions, whilst also minimising bias (errors) of predictions. However, this ‘bias-variance trade-off’ does not ensure the developed model has low instability in the chosen target population, and we can examine this using bootstrapping, as follows.

Assume that a prediction model has been developed using a particular model development process and set of candidate predictors, applied to particular dataset obtained from the chosen target population. To examine the instability of this developed model, a bootstrapping approach can be applied as described in Table 1. It resamples the original data to create $B$ different samples (of the same size), and in each sample a model is developed following exactly the same development process used to produce the model in the original data. The multiverse of $B$ models can then be compared (e.g. in terms of their included predictors) and the variability (instability) in predictions can be quantified at the individual level, as outlined by Riley and Collins [12]. We suggest presenting a *prediction instability plot*: a scatter of the $B$ predicted values for each individual against their predicted value from the original developed model, with uncertainty intervals included (e.g. 95% using the 2.5th and 97.5th percentiles). The mean absolute prediction error (MAPE) can be calculated for each individual [7, 12, 15], which is the mean of the absolute difference (‘error’) between the bootstrap model predictions (${\widehat{p}}_{bi})$ and the original model prediction (${\widehat{p}}_{i})$. The variance of the absolute prediction errors might also be summarised for each individual.

**Table 1:** The bootstrap process to examine instability of model predictions in a chosen target population, as adapted from Riley and Collins [12]

Using the model development dataset of $n$ participants from the chosen target population, we recommend the following process:
• Step 1: Use the developed model to make predictions (${\widehat{p}}_{i})$ for each individual participant ($i=1$ to $n$) in the development dataset
• Step 2: Generate a bootstrap sample with replacement, of size $n$
• Step 3: Develop a bootstrap prediction model in the bootstrap sample, replicating exactly (or as far as practically possible) the same model development approach and set of candidate predictors as used originally
• Step 4: Use the bootstrap model developed in step 3 to make predictions for each individual ($$i)$$ in the original dataset. We refer to these predictions as $${\widehat{p}}_{bi}$$, where $$b$$ indicates which bootstrap sample the model was generated in ($$b$$ = 1 to $$B$$)
• Step 5: Repeat steps 2 to 4 a total of $(B-1$) times, and we suggest $B$ is at least 200
• Step 6: Store all the predictions from the $B$ iterations of steps 2 to 5 together in a single dataset, containing for each individual a prediction (${\widehat{p}}_{i})$ from the original model and $B$ predictions (${\widehat{p}}_{1i} , {\widehat{p}}_{2i} ,\dots , {\widehat{p}}_{Bi})$ from the bootstrap models
• Step 7: Summarise the instability in the predictions. In particular, quantify the mean absolute prediction ‘error’ (MAPE) for each individual, and summarise this across individuals, and display a prediction instability plot (scatter of the $B$ predicted values for each individual against their original predicted value). Other instability plots (e.g. for classification, clinical utility) and measures may also be useful, as shown elsewhere [12].

### Applied examples and the impact of sample size on instability

To illustrate the concepts of the multiverse and instability, we develop prediction models to estimate the risk of 30-day mortality in individuals diagnosed with an acute myocardial infarction (MI). We use the GUSTO-I dataset [16], which includes 40,830 participants from the target population, of which 2851 (7%) died by 30 days. To the data, we fitted a logistic regression model with a lasso penalty [17], considering eight predictors: sex, age, hypertension, hypotension, tachycardia, previous MI, ST elevation on ECG and systolic blood pressure. In the original data, all eight predictors were retained, and the c-statistic was 0.80, with a Nagelkerke R^2^ of 0.21 (21% explained variation). Of interest is whether this model has instability in individual predictions arising from epistemic (reducible) uncertainty in the development process; in contrast, we are not focused on prediction uncertainty due to aleatoric (irreducible) uncertainty (the approximately 79% of remaining outcome variability the model could not explain).

**Fig. 2:**
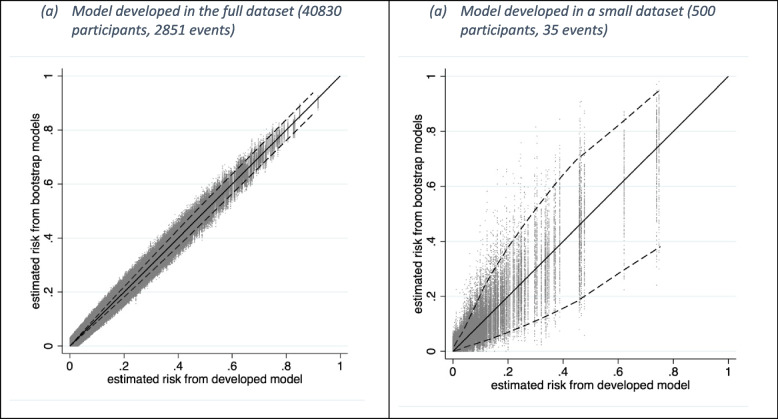
Prediction instability plot for a logistic regression model (with a lasso penalty) considering 8 predictors fitted in **a** the full sample of 40,830 participants (2851 deaths) and **b** a sub-sample of 500 participants (35 deaths). The solid diagonal line indicates perfect agreement between the predictions from the developed model and predictions in the bootstrap model. The vertical spread of points indicates the instability in the multiverse, reflecting differences in an individual’s prediction from the developed model (our universe) and their prediction in other hypothetical models (other universes). The dashed lines denote the 2.5th and 97.5th percentiles of the distribution

Applying the bootstrap process of Table 1, we found that across 500 models (developed in 500 bootstrap samples) there was low variability in individual predictions (Fig. 2a), with an average MAPE across individuals of 0.0028 and a largest MAPE of 0.027. As such, the models in the multiverse all give similar predictions, meaning instability at the individual level is not a concern. This gives strong reassurance that the original developed model is stable in the target population represented by the development dataset. This is expected given the large size of this development dataset, with about 356 events per predictor parameter, far exceeding the minimum sample size of about 7 events per predictor parameter for this scenario based on proposed sample size criteria [18].

We now assume the available data to develop the model is a random sub-sample of the full GUSTO-I dataset comprising 500 participants and 35 deaths, and so provides a substantially smaller sample size. As before, we fit a logistic regression model with a lasso penalty, where again all eight predictors were retained. The c-statistic is marginally higher at 0.82. Researchers might believe this model has promise. However, given the large reduction in sample size, the corresponding multiverse may have substantial instability across the bootstrap models and individual predictions [18, 19]. This is exposed by applying the bootstrap process from Table 1, revealing huge variability in individual predictions (Fig. 2b). For example, an individual with an estimated 30-day mortality risk of 0.2 from the original model has a wide range of alternative predictions from about 0 to 0.8 across the multiverse (compared to 0.15 to 0.25 across the multiverse of models derived from the full sample), potentially reflecting substantial differences in subsequent clinical decisions and treatment choice. Individuals have an average MAPE of 0.023 and a largest MAPE of 0.14 (compared to 0.0028 and 0.027, respectively, across the multiverse of models derived in the full sample). Hence, despite the apparently good discrimination performance, there is large instability in predictions for some individuals. This is anticipated given there are only about 4 events per predictor parameter, much fewer than the minimum of 7 recommended by our sample size criteria [18].

### Why does instability in individual predictions matter in healthcare?

Instability of individual predictions is relevant because, within healthcare, model predictions guide individual counselling, prioritisation of resources, and clinical decision making. If different samples lead to models with very different predictions for the same individual, then this reduces our assurance in using a particular model’s predictions to inform clinical decisions.

**Fig. 3:**
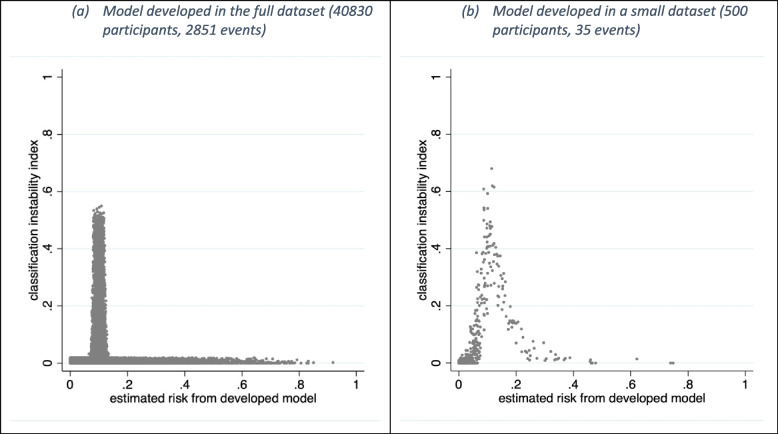
Classification instability plot for logistic regression models with a lasso penalty considering 8 predictors and a risk threshold of 0.1

Suppose a model is to be used for classification, like when a threshold is used to guide clinical decisions; for example, a general practitioner might consider prescribing a statin to an individual if their estimated cardiovascular disease risk is at least 0.1 [20]. An individual enters a multiverse of madness if the developed model (based on a particular sample) suggests their risk is > 10%, but a large proportion of other models from the multiverse (each based on a different sample from the same population) suggests their risk is < 10%. This can be quantified using the *classification instability index*, which estimates an individual’s probability that their prediction (from the original model) leads to a different classification to those from the bootstrap models. Figure 3 shows the classification instability index for the previous models based on a risk threshold of 0.1. In the full dataset, the index is small except for individuals with predictions from the original model very close to the 0.1 threshold. However, when the development sample size is 500 participants, the classification instability index is larger; for example, some individuals with an original prediction of 0.2 have about a 15-20% chance of being classified below the threshold in a different model. This might flag concern about using the original developed model to make classifications in individuals.

Some instability is inevitable and needs to be viewed in context of the problem at hand: is the aim of the model to improve population-level outcomes as a whole, or to ensure the best shared decision making for each individual? In other words, do we care which individuals the model predicts accurately in, or do we want to ensure that the model predictions are accurate in every subgroup of individuals (e.g. defined by one or more characteristics)? This relates to the hierarchy of prediction model calibration proposed by Van Calster et al.; [21] a model’s predictions may be well calibrated at the population level, but not well calibrated when broken down into more refined groupings. Indeed, the latter may be a utopia that is very difficult to achieve unless data are enormous.

A model may still have benefit even with instability at the individual level. For instance, instability in regions of very high risk (e.g. reflected by uncertainty intervals from 0.3 to 1) may not matter if clinical decision thresholds are much lower (e.g. 0.05 to 0.1). Similarly, the decision to prescribe statins to those with an estimated 10-year cardiovascular disease (CVD) risk > 10% may still reduce the CVD rate in the UK population as a whole [22], even with instability at the individual level. This can be gauged by examining stability in a model’s overall clinical utility [12], for example using net benefit [23]. Nevertheless, we should still strive for stability at the individual level, as most healthcare consultations aim to improve individual-level outcomes and so models ideally need to support this. An example is injury prediction in elite sports players, where the goal is to improve outcomes for each individual player [24], not the population of elite sports players per se.

### Instability of individual predictions and impact on discrimination

Individual predictions may be unstable even when there is stability in a model’s discrimination performance, which measures the separation in estimated risks for those with and without the outcome event. In particular, the c-statistic measures the proportion of all pairs (one patient with the outcome event, one patient without the outcome event) where the model estimates a higher risk for the patient with the outcome event. In our aforementioned model developed using 500 participants, applying each bootstrap model to the original sample, 95% of the c-statistic estimates range from 0.77 to 0.82. Thus, the c-statistic appears quite stable in the multiverse, despite the aforementioned huge instability in individual-level predictions. This is because the c-statistic is a summary measure based on all individuals, so is less vulnerable to instability concerns at the individual level.

### Instability of individual predictions and impact on calibration

**Fig. 4:**
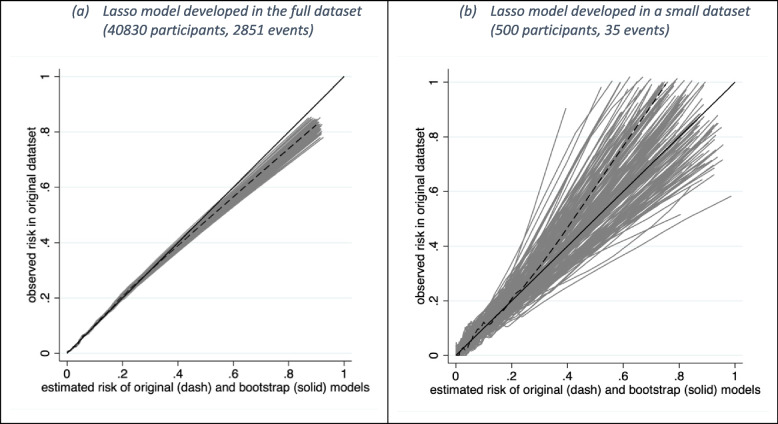
Calibration instability plot for a logistic regression model (with a lasso penalty) considering 8 predictors fitted in **a** the full sample of 40,830 participants (2851 deaths) and **b** a sub-sample of 500 participants (35 deaths). The solid diagonal line indicates ideal calibration. The dashed line indicates the calibration curve of the original model in the original sample. Others are the calibration curves of 200 bootstrap models applied in the original sample

Calibration measures the agreement between observed and estimated risks [25]. A model’s estimated risks are more likely to be miscalibrated in the chosen target population when there is large individual-level instability [6]. This can be exposed by fitting smoothed calibration curves for the bootstrap models applied in the original dataset. These curves can be overlayed together on the same graph to form a *calibration instability plot*. Large variability (instability) in the curves raises a concern that the model predictions will be miscalibrated in the target population. Figure 4 shows the calibration instability plot for the models developed in the full and small datasets. There is very little variability in curves for models developed in the full dataset, but large variability in curves for models developed in the small dataset.

### Do instability concerns apply to AI or machine learning methods?

The potential issue of instability applies irrespective of the underlying modelling approach, including those ascribed to AI or machine learning such as ensemble methods. Ensemble methods already recognise the multiverse by averaging predictions over multiple models in the multiverse, in order to reduce bias and variability (instability) based on any one model alone. For example, random forests are an ensemble method that uses bagging (bootstrap aggregating) to develop multiple forests from multiple bootstrap samples and then average predictions over them. Another example is stacking, also known as super learners, which average predictions across multiple models fitted to the same development dataset. Often the set of models used are deliberately diverse (e.g. in their format and set of considered predictors).

Although such ensemble methods are designed to reduce instability, they do not alleviate it. Similarly, popular methods for penalised regression (e.g. lasso, elastic net, ridge regression) can be very unstable (e.g. see Fig. 2b), even though they are designed to improve models in situations with high variance (instability) that arise in small samples and large numbers of predictor parameters [6, 26, 27]. For example, Riley and Collins show instability of models developed using penalised regression and random forests [12], and demonstrate how instability may depend heavily on the model tuning process (e.g. selecting the number and depth of trees), and increases with the use of data splitting into training and test samples. Ultimately, all methods are limited by the available sample size for model development, unless strong external information is also incorporated (e.g. via informative prior distributions in a Bayesian framework)—though in practice this is rarely done.

### Addressing instability in the multiverse by targeting larger sample sizes

Development datasets with small sample sizes lead to unreliable individual-level predictions from unstable models, caused by uncertainty in the model (e.g. regression equation, tree structure). Therefore, to improve stability when developing a new model, researchers should seek to reduce model uncertainty by adhering to minimum sample size requirements that (i) aim to precisely estimate the overall risk or mean value in the population and (ii) target a small amount of model overfitting [18, 19, 28]. Even then, the instability may be quite pronounced, and substantially larger datasets may be required to sufficiently negate instability concerns for all individuals [7]. Sample size criteria based on precisely estimating predictor effects and targeting low MAPE values (e.g. < 0.02) can help address this [15, 18, 19, 28].

In a situation where participants are still being recruited into a model development study, learning curves may help identify when additional data are needed to reduce current instability levels, for example as identified by instability plots and measures such as MAPE [29], or low effective sample sizes for individuals [30]. If further recruitment is not possible and instability is large, it may simply not be sensible to proceed with model development unless external information (e.g. about predictor effects, tuning parameters) can be borrowed or a different, more stable model development approach chosen.

### Instability checks are important for model fairness and model comparisons

Instability checks can also be used to inform investigations of model reliability in different types of participants, for example by checking MAPE in subgroups defined by ethnicity [12]. There may be more instability in some groups than others, especially for those with a small sample size (e.g. due to being underrepresented or having rare characteristics), and ultimately this may lead to concerns about a model’s fairness if used in practice. Instability checks also help compare competing models, or even model development strategies. For example, if models A and B have similar c-statistics and clinical utility, but model A has greater stability in individual predictions, then it makes sense to use model A.

### Are there limitations with the bootstrap approach?

It is important that the bootstrap process mimics, as closely as possible, the steps taken to develop the prediction model, so that the instability in the model development process is fully captured. This includes any approaches for data splitting, variable selection, missing data handling and model tuning. We appreciate this may be computationally intensive, even more so for deep learning methods. The quality of the bootstrap process in reflecting the instability in the target population is also dependent on the representativeness of the development sample for the target population (e.g. in case-mix variation, the intended moment of prediction and measurement of predictors and outcomes) [31]. Bootstrap samples taken from small and non-representative samples may underestimate the actual instability in the underlying population. Further, evaluations in *other *populations require external validation in new data, with sufficient sample size [32–34], sampled from those other populations.

### Might the multiverse be even more diverse?

So far, we have focused on instability in a multiverse of models that are all developed using the same development approach on the same set of predictors. This is akin to what might happen if we placed the original researchers into each universe, and ensured they develop models using the same protocol, analysis plan, modelling strategy, set of candidate predictors and so forth. However, the multiverse would be even more diverse if we allowed there to be additional uncertainty due to researcher (modeller) preferences, for example in regard to their choice of modelling strategy (e.g. lasso, elastic net, random forest or super learners), methods for handling missing data and sets of candidate predictors. Researchers could examine this by extending the proposed bootstrap process to allow Step 3 to randomly select from a range of other plausible modelling choices, but usually this is not necessary or informative.

Lastly, note that we focused on instability of a developed model for *one* chosen target population. Consideration of *different *(other) target populations (e.g. different settings, countries) is about generalisability and transportability [35], rather than instability of the developed model for the originally intended target population.

## Conclusions

“*The Multiverse is a concept about which we know frighteningly little*”

Dr. Strange (Spider-Man: No Way Home)

Whenever researchers develop a clinical prediction model using a particular dataset and model development approach, we recommend they use bootstrapping to investigate the corresponding multiverse of models and instability of individual predictions. They should report their findings in terms of MAPE and instability plots (e.g. for prediction, classification and calibration), as these add extra information over established performance metrics like R^2^, c-statistic, calibration-in-the-large, calibration slope and net benefit. Clear reporting of instability will help expose whether a model’s predictions are likely reliable at the individual level; enable more informed judgements about the model’s quality (risk of bias [36]) in the context of its potential use; identify whether larger development datasets (or external information) are needed to reduce instability; motivate the use of alternative modelling approaches that improve stability (e.g. better tuning approaches such as repeated rather than single cross validation; exclusion of a few predictors that especially inflate instability); and reveal the need for further validation studies. It may also motivate approaches to take forward the uncertainty in a model’s prediction (e.g. via a predictive distribution from a Bayesian analysis), rather than just using a single predicted estimate for an individual. The forthcoming TRIPOD + AI guideline includes recommendations for reporting any assessment to examine model instability in recognition of its importance and impact on using model prediction to guide decision making [37]. Example code for examining stability is available at www.prognosisresearch.com/software, and stability plots are an option within the *pminternal *R package [38].

## Data Availability

In all our case studies, models are developed using the GUSTO-I dataset that contains individual participant-level information on 30-day mortality following an acute myocardial infarction. The dataset is freely available, for which we kindly acknowledge Duke Clinical Research Institute, and can be installed in R by typing: load(url('https://hbiostat.org/data/gusto.rda')).
